# Quality standards in respiratory real-life effectiveness research: the REal Life EVidence AssessmeNt Tool (RELEVANT): report from the Respiratory Effectiveness Group—European Academy of Allergy and Clinical Immunology Task Force

**DOI:** 10.1186/s13601-019-0255-x

**Published:** 2019-03-27

**Authors:** Nicolas Roche, Jonathan D. Campbell, Jerry A. Krishnan, Guy Brusselle, Alison Chisholm, Leif Bjermer, Mike Thomas, Eric van Ganse, Maarten van den Berge, George Christoff, Jennifer Quint, Nikolaos G. Papadopoulos, David Price

**Affiliations:** 1 Pneumologie, Hôpital Cochin, 27 rue du Faubourg Saint Jacques, 75014 Paris, France; 2 Center for Pharmaceutical Outcomes Reasearch, Skaggs School of Pharmacy and Pharmaceutical Sciences, University of Colorado, Anschutz Medical Campus, Aurora, CO USA; 3 University of Illinois, Champaign, IL USA; 4 Department of Respiratory Medicine, Ghent University Hospital, 9000 Ghent, Belgium; 5 Syneos Health, 10 Bloomsbury Way, London, WC1A 2SL UK; 6 Department of Respiratory Medicine and Allergology, Skane University Hospital, 221, 85 Lund, Sweden; 7 University of Southampton, University Road, Southampton, SO17 1BJ UK; 8 Claude-Bernard Lyon1 University, 43 Boulevard du 11 Novembre 1918, 69100 Villeurbanne, France; 9 University of Groningen, Hanzeplein 1, 9713 GZ Groningen, Netherlands; 10 Medical University - Sofia, Faculty of Public Health, 8 “Bialo more” str, 1527, Sofia, Bulgaria; 11 Imperial College London, South Kensington Campus, London, SW7 2AZ UK; 12 National and Kapodistrian University of Athens, Fidippidou 41 Str, Goudi, Athens, 11527 Greece; 13 Observational and Pragmatic Research Institute, 60 Paya Lebar Road, Paya Lebar Square, #05-33/34, Singapore, 409051 Singapore

**Keywords:** Asthma, Comparative effectiveness, Quality standards, Observational studies, Database

## Abstract

**Introduction:**

A Task Force was commissioned jointly by the European Academy of Allergy and Clinical Immunology (EAACI) and the Respiratory Effectiveness Group (REG) to develop a quality assessment tool for real-life observational research to identify high-quality real-life asthma studies that could be considered within future guideline development.

**Methods:**

The resulting REal Life EVidence AssessmeNt Tool (RELEVANT) was achieved through an extensive analysis of existing initiatives in this area. The first version was piloted among 9 raters across 6 articles; the revised, interim, version underwent extensive testing by 22 reviewers from the EAACI membership and REG collaborator group, leading to further revisions and tool finalisation. RELEVANT was validated through an analysis of real-life effectiveness studies identified via systematic review of Medline and Embase databases and relating to topics for which real-life studies may offer valuable evidence complementary to that from randomised controlled trials. The topics were selected through a vote among Task Force members and related to the influence of adherence, smoking, inhaler device and particle size on asthma treatment effectiveness.

**Results:**

Although highlighting a general lack of high-quality real-life effectiveness observational research on these clinically important topics, the analysis provided insights into how identified observational studies might inform asthma guidelines developers and clinicians. Overall, RELEVANT appeared reliable and easy to use by expert reviewers.

**Conclusions:**

Using such quality appraisal tools is mandatory to assess whether specific observational real-life effectiveness studies can be used to inform guideline development and/or decision-making in clinical practice.

**Electronic supplementary material:**

The online version of this article (10.1186/s13601-019-0255-x) contains supplementary material, which is available to authorized users.

## Introduction: background and objectives

Randomised controlled trials (RCTs) are required to establish the efficacy and explore the safety of asthma treatments, but are insufficient to provide holistic evidence on the benefit/risk ratio of interventions when offered, initiated or used in the heterogeneous asthma patient populations and care settings that exist outside the trial environment. Registration asthma and allergy RCTs are designed to maximize chances of observing efficacy [1]. To do so, they impose a standardised level of care (often more intensive and extensive than is feasible in routine care) and systematically exclude patients presenting with characteristics that could modulate treatment responses such as smoking, obesity, poor adherence, inhaler misuse, poor reversibility, possible overlap with chronic obstructive pulmonary disease (COPD) or other comorbidities [2–7]. In this way, registration RCTs guarantee high internal validity to obtain market authorization of the drug but do not provide sufficient evidence to guide decision-making in routine clinical practice, since they exclude a significant proportion of the real-life asthmatic population [8]. Excluded patients may also represent subsets of the population in whom achieving asthma control is more difficult, i.e. those who warrant increased attention and monitoring. Therefore, it is necessary to test the external validity of the findings of the registration RCTs: their generalizability and their applicability to the general asthma population and to specific subgroups usually excluded from RCTs [9]. It is important to find reliable research methods to complement RCTs and broaden the evidence-base available to inform clinical practice decision-making [10–12]. Several study designs are available to reach this goal, including pragmatic randomized trials and observational studies, which can be prospective or historical, the latter relying on clinical cohorts, registries or databases [1, 13]. Phase IV comparative effectiveness studies are crucial not only to clinicians, but also to other stakeholders such as guideline developers and healthcare policy makers [14]. They can also generate new hypotheses to be tested in RCTs or other adapted study designs.

**Fig. 1:**
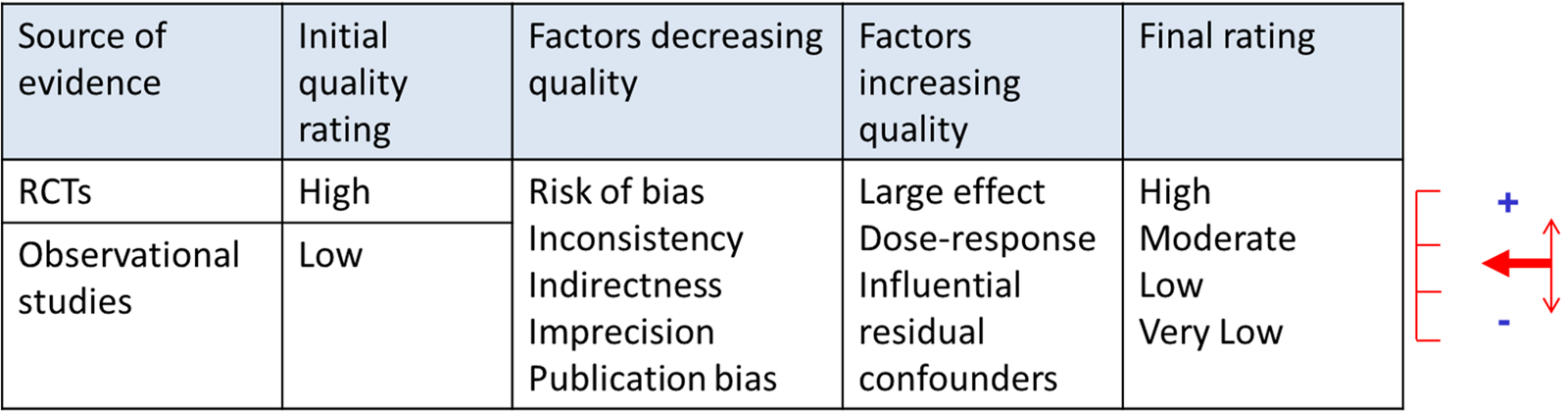
GRADE assessment of the strength of evidence of individual studies and systematic reviews. Adapted from [18]

In observational research as well as in RCTs, ensuring high-quality methodology is crucial to avoid biases that would compromise the reliability and validity of results [15]. Following the GRADE methodology for evidence appraisal, RCTs are initially considered as providing high levels of evidence while observational studies are set at a lower level [16, 17]. Accounting for the quality of available studies, which determines the risk of biases, levels of evidence can be downgraded for RCTs and upgraded for observational research (Fig. 1) [18]. However, while quality standards for RCTs are well-defined and extensively disseminated, e.g., by the CONSORT statements [19, 20], this is less so in the field of observational comparative effectiveness research.

**Fig. 2:**
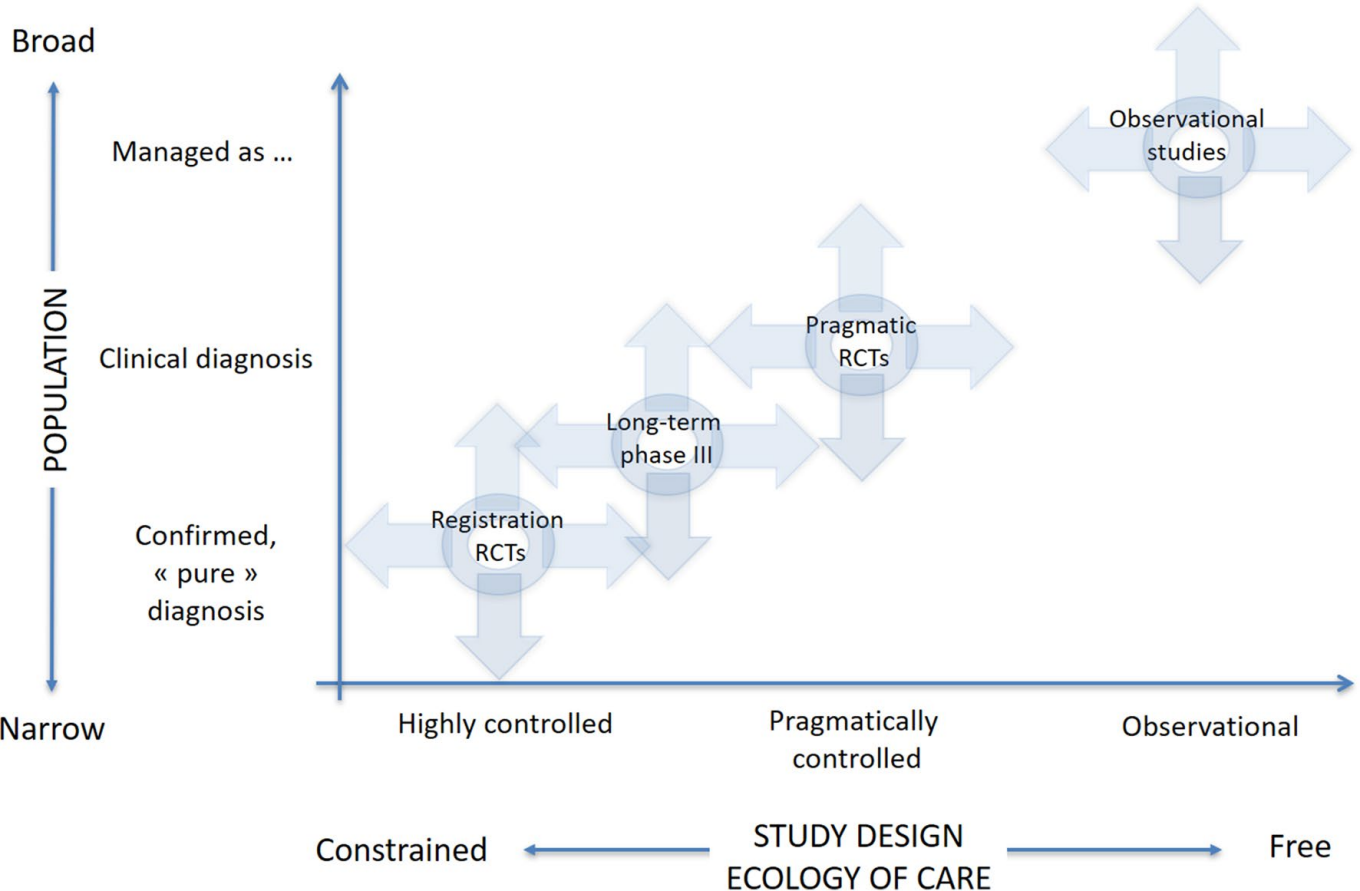
REG research framework. Reproduced with permission from [1]

A number of frameworks have been proposed to help characterise the extent to which a study design reflects pragmatic aspects or real-life patients and practice [1, 21]. One of these frameworks (developed by respiratory clinicians but more generally applicable) relies on two axes, namely the population’s characteristics and the so-called “ecology of care”, which encompasses the context in which care is delivered (e.g., routine primary care vs. controlled conditions with regular visits to specifically trained healthcare professionals, as in most registration RCTs) (Fig. 2) [1]. This framework was proposed by the Respiratory Effectiveness Group (REG), an academic non-profit organization created in 2012 with the aim of promoting high-quality real-life research in respiratory diseases. Several tools to guide the design and reporting of observational research have also been developed, sometimes using systematic and rigorous processes [22–27]. While these provide invaluable insights and recommendations for evidence generation, they have not been specifically designed for the evaluation of published (comparative) effectiveness research.

To address this, REG and the European Academy of Allergy & Clinical Immunology (EAACI) convened a joint Task Force to set and test quality standards for observational comparative effectiveness research (CER) in asthma, and to assess the extent to which the evidence from such studies might complement the results of RCTs. The aim of the current manuscript is to provide an overview of how this Task Force developed a quality assessment tool and applied it to selected PICOT questions for which RCTs provided only limited evidence. The purpose of this paper is not to provide an extensive description of the tool development process, nor the detailed results of the literature review; these will be the topics of additional publications (see [28] for details on the tool's development).

## Methods

The initial work comprised two simultaneous processes: [1] the development of a quality assessment tool through a synthesis of Task Force expertise and recommendations within existing relevant publications and [2] the selection of relevant research topics and identification of corresponding articles that would be used to test the tool. Once the tool was finalised (following pilot testing and iterative refinement), it was used to appraise the retrieved literature. Studies achieving sufficiently high quality scores were then assessed to determine the extent to which they offered novel data and insights that could influence clinical practice and inform guidelines. A Task Force activity schedule summary is presented in the Additional file 1: Table S1.

### Development and testing of the quality assessment tool

Full details of the REal Life EVidence AssessmeNt Tool (RELEVANT) development and assessment process are detailed in a dedicated article [28]. In summary, a systematic review of related quality tools and recommendations for effectiveness research proposed in the published literature was performed [22–27]. Appropriate quality assessment domains and specific sub-items were identified, agreed through Task Force discussions and used to build a first version of RELEVANT. The domains were: background, study design, measures, analysis, presentation of results, discussion and interpretation, conclusions and reporting of possible conflicts of interest including, but not restricted to, study funding. For each domain, items (n = 25 altogether) were divided into primary and secondary/supporting. The resulting assessment grid was then tested by 9 members of the Task Force divided in two groups, each assessing three articles selected by the Task Force leads. For each item, the agreement between raters was calculated before being summarized for all primary items, all secondary items and all (primary and secondary) items combined. Three of the papers reviewed considered the relationship between adherence and outcomes in patients with asthma, and three papers examined the relationship between particle size or device type and asthma outcomes [29–34]. Rater comments on the ease of implementation of the RELEVANT tool were collected as free text using a dedicated table. Levels of agreement and rater comments were discussed face-to-face at a Task Force meeting and the tool was refined and formatted to be usable on a web-based platform. The resulting version of the tool was tested on a larger basis by 22 raters distributed into 3 groups reviewing 2 papers each. There were two papers on the relation between adherence and asthma outcomes, two on the relation between drug persistence and healthcare resource utilization and two on the relation between particle size and outcomes [35–40]. Additional free-text feedback was captured and used to guide further refinements and to finalise the first version of RELEVANT.

### Selection of topics and literature search

**Fig. 3:**
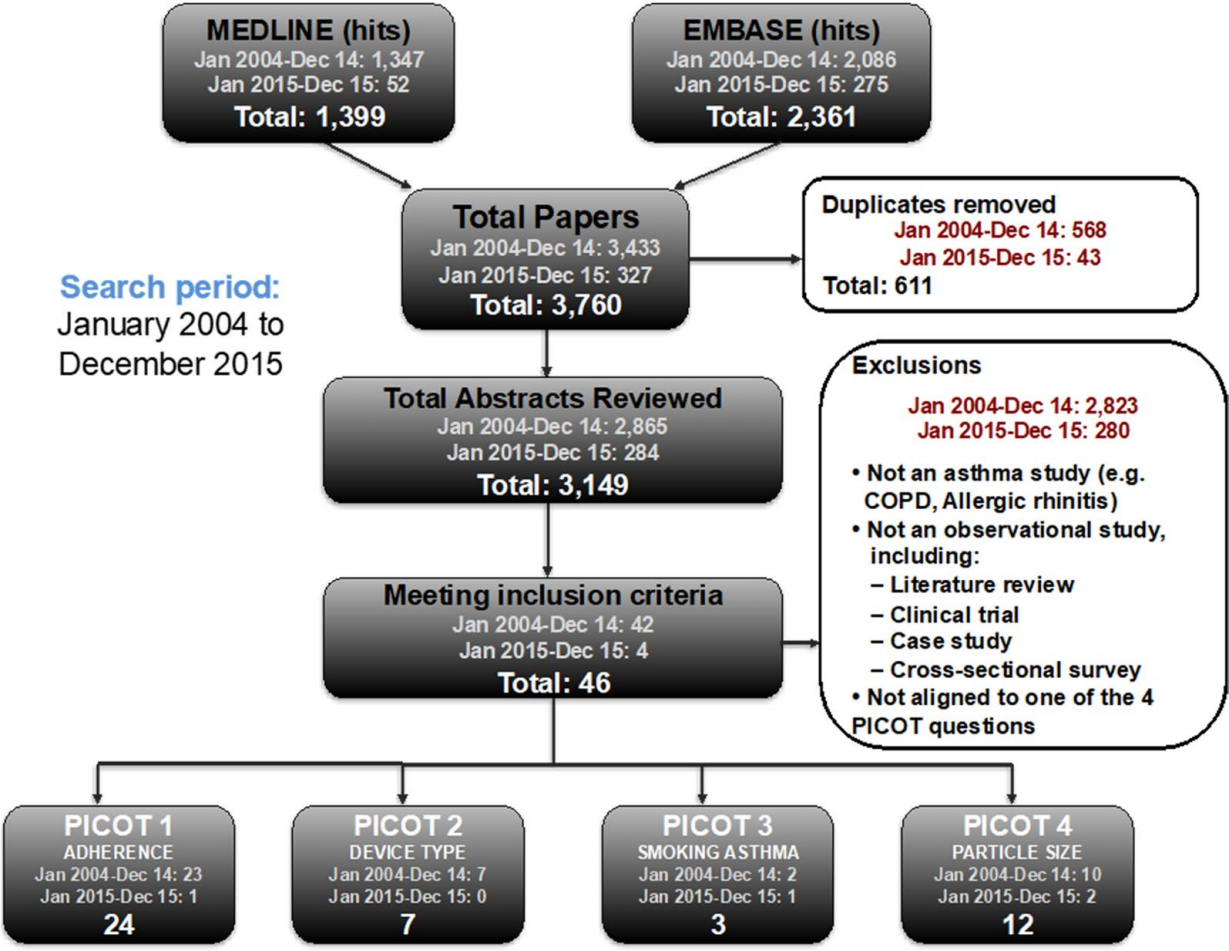
Flow diagram for inclusion/exclusion of articles

A first set of 21 PICOT [(P)opulation selected for the study, (I)ntervention, (C)omparator, (O)utcome measures and (T)ime—duration] [41] questions was identified during a Task Force meeting. The questions were selected based on their relevance for asthma management, limited ability of available RCTs to provide robust answers and potential of observational research to address them. Discussions in a plenary session led to the prioritization of 9 PICOT questions. An online poll was conducted among REG members and EAACI representatives to prioritise the topics and 4 PICOT questions were finally selected to ensure feasibility of delivery. The literature search was conducted using the Medline and EMBASE bibliometric databases to identify asthma effectiveness studies. Search results were then categorised to relevance to the selected PICOT questions (see the Additional file 1 for details on the search strategy, Table S2). The flow diagram shown in Fig. 3 depicts the main steps of the selection process.

### Assessment of the literature

Papers considered of sufficient quality were sent to at least two raters each with a reading grid (see Additional file 1: Table S3) comprising the following items: summary of methods (studied population, intervention, outcomes, setting), summary of results (magnitude of differences/robustness) and possible remaining biases. The list of raters is available in the Additional file 1: Table S4. Each paper was sent to a third rater when there was discordance in the conclusions from the two initial raters. For each PICOT question, a summary table was built, mentioning, for each article, the type of source (e.g. claims database or prospective cohort), the main conclusion, the corresponding level of evidence proposed based on GRADE assessment, the possible impact on clinical practice and whether similar evidence was available from RCTs. Summary tables were presented and discussed during a dedicated plenary session at the REG Summit in June 2016.

## Results

### RELEVANT: the REal Life EVidence AssessmeNt Tool

RELEVANT is presented in Table 1. For each quality domain, sub-items are categorised as primary or secondary items. The general principle for use of the tool is that a study should only be eligible to inform guideline development (or a similar processes) if all primary items are satisfied. Thereafter, secondary items can be used to further appraise non-essential, but pertinent aspects of published studies. Thus, the tool exists in two formats: [1] a version specific to guideline development, in which secondary items appear to the rater only when all primary quality items are satisfied, and [2] a version for more general quality appraisal in which all primary and secondary items are immediately visible.

**Table 1:** RELEVANT REG quality assessment tool for observational research

Primary items	
1. Background	1.1. Clearly stated research question
2. Design	2.1 Population defined
	2.2. Comparison groups defined and justified
3. Measures	3.1. (If relevant), exposure (e.g. treatment) is clearly defined
	3.2. Primary outcomes defined
4. Analysis	4.1. Potential confounders are addressed
	4.2. Study groups are compared at baseline
5. Results	5.1. Results are clearly presented for all primary and secondary endpoints as well as confounders
6. Discussion/interpretation	6.1. Results consistent with known information or if not, an explanation is provided
	6.2 The clinical relevance of the results is discussed
7. Conflict of interests	7.1. Potential conflicts of interest, including study funding, are stated
Secondary items	
1. Background	1.1. The research is based on a review of the background literature (ideal standard is a systematic review)
2. Design	2.1. Evidence of a priori design, e.g. protocol registration in a dedicated website
	2.2 Population justified
	2.3 The data source (or database), as described, contains adequate exposures (if relevant) and outcome variables to answer the research question
	2.4 Setting justified
3. Measures	3.1 Sample size/Power pre-specified
4. Analysis	*NO SECONDARY ITEMS*
5. Results	5.1. Flow chart explaining all exclusions and individuals screened or selected at each stage of defining the final sample
	5.2. The authors describe the statistical uncertainty of their findings (e.g. p-values, confidence intervals)
	5.3. The extent of missing data is reported
6. Discussion/interpretation	6.1. Possible biases and/or confounding factors described
7. Conflict of interests	*NO SECONDARY ITEMS*

The final list of 21 items within RELEVANT was informed by appraisal of inter-rater agreement. Initial agreement was poor (≤ 50%) for 8/25 items when first piloted among Task Force members. Iterative refinement was then based on rater feedback and removal of ambiguity in time language to avoid potential for uncertainty or differing interpretation. This resulted in a marked improvement in concordance between raters, to ≥ 73% for all primary and ≥ 69% for all secondary items when averaged over the three rating groups. Further details on concordance are available in the accompanying methodology paper [28].

### PICOT questions

The four selected PICOT questions (numbered following their rank during the selection process) dealt with the influence of adherence/persistence on asthma outcomes, the influence of smoking on asthma outcomes and treatment effectiveness, the impact of the inhaler device on asthma outcomes and the relation between particle size of maintenance therapy and asthma outcomes (Table 2).

**Table 2:** Selected PICOT questions

Question	Influence of adherence to ICS therapy on asthma outcomes	Influence of device type for ICS therapy on asthma outcomes	Influence of smoking on asthma outcomes in patients receiving ICS therapy	Influence of ICS particle size on asthma outcomes
Population	Asthmatics of all ages prescribed regular maintenance ICS	Asthmatics of all ages prescribed regular maintenance ICS	Asthmatics of all ages prescribed regular maintenance ICS	Asthmatics of all ages prescribed regular maintenance ICS
Intervention	Adherence to recommended therapy	Different inhaler devices/delivery systems	Smokers	Extra-fine particles ICS
Comparison	Different levels of adherence (e.g. 0–25%, 25–50%, 50–75%, over 75%)	Different inhaler systems (pMDI, breath-activated MDI, DPI) for delivering the same molecule	Ex/non-smokers	Fine particles ICS
Outcomes	Exacerbations, admissions, symptoms, QOL	Exacerbations, admissions, symptoms, QOL	Exacerbations, admissions, symptoms, QOL	Exacerbations, admissions, symptoms, QOL
Time frame	≥ 12 months	≥ 12 months	≥ 12 months	≥ 12 months

### Selected articles

**Fig. 4:**
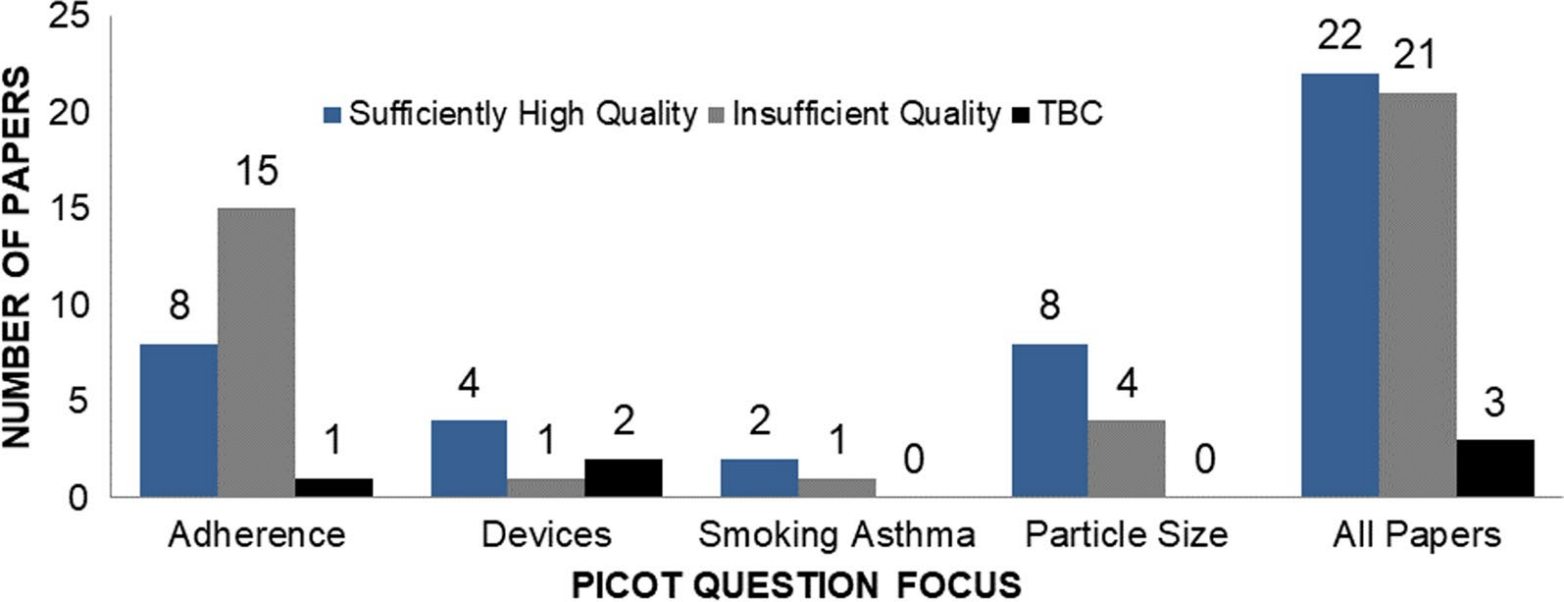
distribution of papers by quality rating within selected PICOT questions. TBC refers to a minority of papers that got only one rating, so that final rating remained to be consolidated

**Fig. 5:**
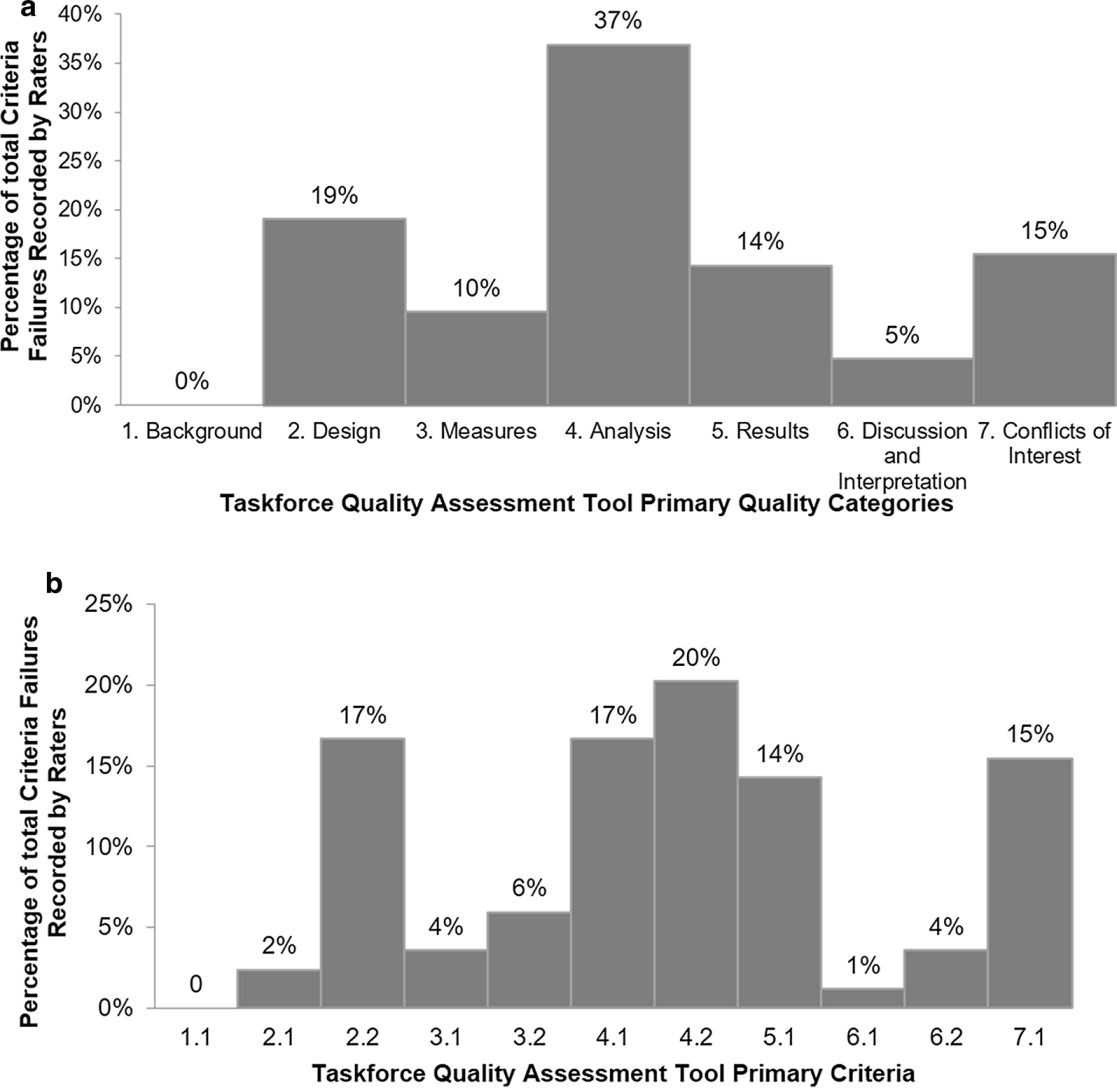
proportion of failed items by domains (**a**) and items **b** of the quality assessment tool (see Table 1 for details on the items’ labelling)

Altogether, 46 relevant articles were identified: n = 24 for PICOT question 1 [7, 29, 35, 36, 38, 42–59], 7 for PICOT question 2 [34, 39, 60–64], 3 for PICOT question 3 [33, 65, 66] and 12 for PICOT question 4 [32, 40, 67–76]. Figure 4 shows the results of the quality assessment of all papers by PICOT question and Fig. 5 provides a summary of failed items (Fig. 5a) and domains (Fig. 5b). The detailed quality assessment of all selected articles is presented in the Additional file 1: Table S5.

### Literature analysis

A summary of the literature analysis is presented in Tables 3, 4 and 5. Study results are presented in a concise way due to space limitations and since more details would actually be out of the scope of the present manuscript. Nonetheless, the main limitation of currently available clinical or claims database studies was found to be the paucity or lack of detailed clinical data regarding: [1] asthma control as assessed by validated questionnaires, [2] severity as assessed by symptoms and lung function, [3] comorbidities, as evidenced by either symptoms suggestive of comorbidities such as rhinitis or gastro-oesophageal reflux disease, or physician diagnoses of comorbidities. As a consequence, authors used available proxy measures, including: [1] short-acting beta-agonist (SABA) use and exacerbations as markers of control; [2] inhaled corticosteroid (ICS) dose and add-on treatments for severity, and [3] prescribed therapies in place of comorbidity diagnoses. In most retained studies, these factors were used to match and/or adjust analyses, a required feature to increase the robustness of results. Use of such statistical approaches allowed the level of evidence to be raised to moderate but not high as residual confounding by hidden factors could not be ruled out; for instance, detailed data on lifestyle behaviours (such as smoking, diet and physical activity) are frequently lacking and body mass index is absent in several databases. The results of observational studies considered of sufficient quality (all 11 primary items fulfilled) suggest several conclusions regarding the corresponding PICOT questions.

**Table 3:** Summary table of literature analysis, PICOT question 1: influence of adherence to ICS therapy on asthma outcomes

Reference	Statement	Type of data source	Final level of evidence (see Fig. 1)	Possible impact on clinical practice (TF opinion)	Similar evidence available from RCTs
Williams et al. [45]	Low adherence increases the risk of ED visits and oral steroid treatment	D-M	Moderate	Yes	No
Taegtmeyer et al. [46]	Lower ACQ improvement associated with low adherence	PC-A	Moderate	Yes	No
Laforest et al. [47]	Low adherence (MPR) associated with poorer control and more hospital contacts and oral steroid courses	PC-A	Moderate	Yes	No
Laforest et al. [48]	Low adherence (MPR) increases the risk of oral steroid treatment and hospitalization	D-A	Moderate	Yes	No
Sadatsafavi et al. [31]	Risk of asthma-related hospitalization lower with ICS-containing regimen than LABA alone	D-M	Moderate	Yes	No
	Risk of asthma-related hospitalization similar between ICS and ICS-LABA	D-M	Moderate	Yes	No^a^
	Risk of asthma-related hospitalization increases when ICS treatment is irregular	D-M	Moderate	Yes	No
Friedman et al. [43]	Adherence and SABA use are better with MF than FP DPIs, with no difference in other clinical outcomes	D-M	Moderate	No	No
Campbell et al. [44]	Shifting drug costs to patients decreases adherence and impairs asthma outcomes	D	Moderate	Yes	No
Tan et al. [38]	In adherent patients, ICS > LTRA	D + S	Moderate (D), low (S)	Yes	In part (pragmatic RCT)
	In non-adherent patients, ICS < LTRA	D + S	Moderate (D), low (S)	Yes	In part (pragmatic RCT)

*SABA* short-acting beta2 agonist, *LABA* long-acting beta2 agonist, *ICS* inhaled corticosteroid, *LTRA* leukotriene-receptor antagonist, *MF* mometasone furoate, *FP* fluticasone propionate, *D* database, *PC* prospective cohort, *S* survey, *M* matched, *A* adjusted, *RCT* randomized controlled trial, *MPR* medication possession ratio, *ED* emergency department, *ACQ* asthma control questionnaire, *TF* task force

^a^Opposite finding regarding the risk of severe asthma exacerbation in several trials

**Table 4:** Summary table of literature analysis, PICOT question 2: influence of device type for ICS therapy on asthma outcomes

Reference	Statement	Type of data source	Final level of evidence (see Fig. 1)	Possible impact on clinical practice (TF opinion)	Similar evidence available from RCTs
Price et al. [60]	One single device for maintenance treatment is better than mixed devices in terms of control and severe exacerbations	D-A	Moderate	Yes	No
Thomas et al. [62]	Switching devices (DPI to pMDI or BAI or other DPI -BAI to pMDI or other BAI) is associated with poorer outcomes	D-M-A	Moderate	Yes	No
Price et al. [61]	pMDI > DPI to administer FP/SAL, in terms of asthma outcomes	D-M-A	Moderate	Uncertain (cause?)	No
Price et al. [34]	BAI and DPI better than pMDI for several asthma outcomes	D-A	Moderate	Uncertain (cause?)	No

*DPI* dry powder inhaler, *BAI* breath-actuated inhaler, *pMDI* pressurized metered-dose inhaler, *FP* fluticasone propionate, *SAL* salmeterol, *D* database, *M* matched, *A* adjusted, *TF* task force

**Table 5:** Summary table of literature analysis, PICOT question 3: influence of smoking on asthma outcomes in patients receiving ICS therapy

Reference	Statement	Type of data source	Final level of evidence (see Fig. 1)	Possible impact on clinical practice (TF opinion)	Similar evidence available from RCTs
Brusselle et al. [33]	Lower asthma control in smokers but same treatment benefit irrespective of smoking status	PC	Low (see text)	No	No
Roche et al. [65]	Better outcomes with extra-fine versus standard size particle ICS, larger differences in current and ex-smokers	D-M-A	Moderate	Uncertain (exploratory)	No

#### Influence of adherence to ICS therapy on asthma outcomes (Table 3) [38, 42–49]

All papers retained for analysis following RELEVANT-based quality assessments reported an association between lower adherence to maintenance therapy and poorer control, as assessed by SABA use or markers of exacerbations (e.g. oral corticosteroid treatment or hospital contacts: emergency room visits, hospitalizations). More specific findings relating to drug classes were reported: oral leukotriene receptor antagonist (LTRA) therapy appeared to be more effective than ICS in obtaining control in non-adherent patients; the opposite was true for adherent patients [38]. Some aspects included in the PICO question could not be addressed: although several papers used the medication possession ratio (MPR; the ratio of the period covered by drug dispensations by the total observation period) to assess level of adherence [36, 47, 50, 51, 77], they did not determine a minimal threshold of adherence below which asthma outcomes worsen. In addition they were not designed to assess whether a “dose–response” relationship could be described.

#### Influence of device type for ICS therapy on asthma outcomes (Table 4) [34, 39, 60–63]

Papers dealing with device choice suggested that limiting the number of different inhaler device types prescribed to an individual patient was associated with better asthma outcomes, while switching devices outside the context of a dedicated medical visit was associated with a greater risk of a loss of control [60, 62]. One study found superiority of a metered dose inhaler (MDI) over a dry powder inhaler (DPI) for the administration of ICS/LABA fixed-dose combination [61]. However, another study by the same group found breath-actuated metered dose inhalers and DPIs to be superior to MDIs for the administration of ICS for several asthma outcomes [34].

#### Influence of smoking on asthma outcomes in patients receiving ICS therapy (Table 5) [33, 65]

A prospective cohort study did not find differential treatment effects of an ICS-LABA fixed dose combination for patients with different smoking behaviours, but asthma patients with a current smoking status had poorer outcomes overall [33]. Another study suggested that extra-fine ICS could be more suitable to control airways inflammation in smokers and ex-smokers than larger particle ICS alternatives [65].

#### Influence of ICS particle size on asthma outcomes (Tables 6, 7) [32, 40, 67–72]

Several analyses, mostly by the same group [32, 40, 67–71], were performed in various populations (children, adults) using various databases (UK, US) and found superiority of extra-fine versus larger particle ICS administered alone or as part of an ICS-LABA fixed dose combination in asthma [32, 40, 67–72]. Accordingly, economic analyses using these results suggested extra-fine ICS was a dominant strategy as compared with larger particle ICS alternatives [32, 69].

**Table 6:** Summary table of literature analysis, PICOT question 4: influence of ICS particle size on asthma outcomes

Reference	Statement	Type of data source	Final level of evidence (see Fig. 1)	Possible impact on clinical practice (TF opinion)	Similar evidence available from RCTs
Van Aalderen et al. [67]	See Table 7	D-M-A	Moderate	Yes	No
Martin et al. [68]	See Table 7	D-M-A	Moderate	Yes	No
Colice et al. [69]	See Table 7	D-M-A	Moderate	Yes	No
Price et al. [70]	See Table 7	D-M-A	Moderate	Yes	No
Price et al. [71]	See Table 7	D-M-A	Moderate	Yes	No
Barnes et al. [40]	See Table 7	D-M-A	Moderate	Yes	No
Price et al. [32]	See Table 7	D-M-A	Moderate	Yes	No
Allegra et al. [72]		PC-A	Low (selection bias, secondary objective)	No	No

**Table 7:** PICOT question 4: influence of ICS particle size on asthma outcomes: summary of results of matched database studies

Reference	Outcomes	Treatments	Population	Database	Results
Van Aalderen et al. [67]	Clinical	BDP pMDI St versus EF Initiation Step-up Vs LABA	Children 5–11	UK (CPRD) US (Optuminsight)	EF > St EF = adding LABA
Price et al. [70]	Clinical	BDP pMDI St versus EF Initiation Switch	12–80	UK GPRD CPRD	EF > St
Price et al. [71]	Clinical	pMDI St FP versus EF BDP Initiation Step-up	5–60	UK GPRD	EF ≥ St at lower doses
Barnes et al. [40]	Clinical	BDP pMDI St versus EF Initiation Step-up	5–60	UK GPRD	EF > St
Martin et al. [68]	C-E	BDP/FP pMDI St versus EF Initiation	12–60/12–80	UK/US	EF dominant
Colice et al. [69]	C-E	pMDI St FP versus EF BDP Initiation Step-up	12–80	UK/US	EF ≥ St at lower doses and costs
Price et al. [32]	C-E	St FP-SAL versus EF BDP-FOR	18–80	UK GPRD CPRD	EF ≥ St at lower doses EF dominant

*EF* extra-fine, *St* standard size, *BDP* beclomethasone dipropionate, *SAL* salmeterol; *FOR*, formoterol, *FP* fluticasone propionate, *pMDI* pressurized metered-dose inhaler

## Discussion

The REG-EAACI Task Force on quality standards in asthma comparative effectiveness research defined target quality standards and developed a quality assessment tool—RELEVANT—to appraise published observational effectiveness studies. The definition of standards and development of the tool aimed to provide a simple assessment grid for paper-based or web-based use, specifically designed for the evaluation of published effectiveness research. They were based on previous initiatives in this area [22–27] and iterative testing and refinement. RELEVANT follows a two-step process: first assessing primary, critical items; then appraising secondary, beneficial quality items. When used in the context of decision-making or guidelines development, it is strongly suggested to stop the assessment and discard the study if any one primary criterion is not satisfied. One major challenge in that respect is to distinguish between a methodological flaw and insufficient reporting. The latter can be overcome (e.g. by contacting the authors for clarifications), but may also be improved through awareness of the RELEVANT tool.

Four PICOT questions were selected through a poll among the Task Force and members of REG and EAACI, and related observational studies identified through a systematic literature search among bibliometric research databases. Retrieved records were then quality appraised using the RELEVANT tool. Several of the papers were assessed as being of sufficiently good quality to allow an increase in their level of evidence from low to moderate following a GRADE-like process. For all PICOT questions of interest, assessed observational studies yielded results with possible impact on clinical practice in areas where similar evidence from RCTs is lacking. Altogether, this suggests that RELEVANT could become a useful tool to appraise the evidence from observational studies as part of a guideline development process as well as offering wider utility for more general literature reading and appraisal, and to inform research development.

### Relevant

A quality assessment instrument needs to offer ease of use and sufficient robustness to ensure confidence in its grading. This requires the items to be clear, easily understood and rated similarly by different raters. While the detailed tool development process is the topic of a dedicated paper [28], it can be emphasized here that the final tool was considered easy to use by members of the REG and EAACI networks who were involved in the final phase. Reaching a 100% inter-rater agreement is probably not realistic considering the heterogeneity of methodology reporting in published papers, but fair levels of agreement were obtained in the implementation phase (≥ 73% for primary items and ≥ 69% for secondary ones averaged over the three raters).

### PICOT questions

An interesting observation permitted by this process was the high number of PICOT questions (n = 21) identified during the first round of selection by the Task Force and its REG and EAACI correspondents. This outlines the evidence gaps that are still present regarding asthma treatment, despite the considerable research interest for this common but still insufficiently controlled disease. This also contrasts with the high number of RCTs assessing the efficacy of various components of asthma care, including both medications and non-pharmacological approaches such as education or integrated care. This discrepancy illustrates that classical RCTs are not sufficient to answer all questions regarding real-life effectiveness of therapeutic strategies, and need to be complemented by dedicated studies designed to reflect better the range of patients and care settings that exist in routine clinical practice [1, 9, 13, 14].

### Literature search: rationale and quantitative results

The initial steps of the process confirmed that conducting a search specific to observational effectiveness research is challenging given the lack of standardised and specific terminology/nomenclature and requires the input of specialists of bibliometric research databases. Despite this, the number of published articles available for each of the PICOT questions was surprisingly low given the long period that was scrutinized (10 years), especially for PICOT questions 2 (influence of device type on the effectiveness of maintenance ICS therapy, n = 7 papers) and 3 (influence of current active smoking on the effectiveness of ICS-containing maintenance therapy regimen, n = 3 papers). This illustrates that observational effectiveness research in asthma is still infrequently performed or reported despite the clear need for real-life data to complement classical RCTs by answering different study questions including to, better assess the extent to which their results can be generalised, and identify the best target populations. Inhalation technique is known to be poor overall with at least 50% of the patients making manipulation and/or inhalation errors, which is associated with poor control of asthma and chronic obstructive pulmonary disease (COPD) [6, 7, 78–80]. This has led to the development of new devices aiming at facilitating the use of inhaled therapy [81]. However, none of these devices can be considered as ideal [82], making it necessary to determine whether some are more effective than others in real-life populations, as opposed to RCTs populations in which patients are specifically educated to use studied devices properly. Regarding smoking asthmatics, in vitro studies showed that smoking decreases the sensitivity of inflammatory cells to corticosteroids, at least in part through oxidative stress-induced decreases in the cofactor histone deacetylase 2 [83, 84]. RCTs also showed a decreased clinical effect of ICS in smoking asthmatics [4]. How this translates within real-life populations is largely unknown. Altogether, real-life effectiveness studies might be useful to better identify subpopulations with specific responses to therapy, allowing more effective treatment individualisation.

The number of articles published in the 10-year period of interest regarding the influence of adherence and particle size on the effectiveness of maintenance ICS therapy was higher than for the two previous topics mentioned above. However, it remained insufficient given the importance of these issues. More specifically, although poor adherence has been associated with both poor inhaler technique and poor disease outcomes [78, 85], the level of adherence required to achieve better control is difficult to determine from RCTs. RCT-reported adherence is believed to be markedly higher than in routine care, although even relatively small differences can be associated with marked differences in outcomes [86]. The search strategy retrieved 24 articles on this topic. While it has been shown with beclomethasone dipropionate that smaller particle size allows a decrease in the nominal dose required to achieve a given physiological effect [87], whether this translates into an improved benefit-risk ratio is unknown, and could be influenced by inhaler technique: the efficacy of extra-fine particle aerosols seems to be less dependent on inhalation technique [88, 89]. Only real-life studies where patients use inhalers with their “natural” technique could help answering this question. Twelve such studies were found to inform PICOT question 4.

### Quality assessment

A common limitation of analysed papers was the lack of precise clinical data available to ensure that patients of compared groups were 100% comparable at baseline. To minimize the impact of confounding (bias), most studies used statistical strategies including matching (exact or propensity score-based) and adjustments for variables differing between groups during the baseline period. These strategies could not, however, account for variables that were entirely absent from the databases. For instance, in most studies lung function data (e.g., FEV1) or questionnaire-measured level of asthma control were not available; so proxies for control were used although they were not necessarily strictly concordant with questionnaire results, e.g., due to time frame differences. In addition, some studies did not report sufficient detail on baseline population characteristics, making it difficult to assess their quality with a high level of confidence. These points reflect the lack of 100% concordance between raters, as outlined above. To deal with this difficulty, a third rater intervened in all cases of discordance between the two initial raters. Ultimately, 48% (see Fig. 4) of eligible papers were considered of sufficient quality to be analysed further in terms of their results and how these could inform current knowledge and guideline recommendations.

There was a marked “research team” bias since 16 articles (34.8%) came from a single research team (references [32, 34, 40, 58, 60–65, 67–71, 76] for the 4 PICOT questions, respectively), among which only 3 (18.8%) [58, 64, 76] were rejected based on quality assessment (vs. 52% of all articles). To avoid possible reviewing bias raters of a given paper could not belong to or collaborate with the research team(s) of the authors. Thus, this observation suggests the importance of experience in the field of observational research to increase the likelihood of high-quality methods.

### Literature analysis

As already outlined, evidence from real-life effectiveness research can complement RCTs in several ways [1, 9]. First, by determining whether results of RCTs are applicable to broader populations receiving usual care, or to populations excluded from RCTs. Second, by exploring whether some subgroups may respond better than others, or could be at increased risk of side-effects. Third, by raising hypotheses on treatments effects or differences unsuspected in RCTs. The PICOT questions selected by the Task Force addressed several of these general issues: PICOT questions 1 and 3 (adherence and smoking) dealt with specific populations usually excluded from RCTs, i.e., poorly adherent patients and smokers, while the two other PICOT questions (2 and 4) dealt with possible differences between treatments that could not be convincingly demonstrated in RCTs. Corresponding hypotheses are that some devices may be associated with better outcomes due to improved ease-of-use or patient’s preference, leading to better adherence (PICOT question 2), and that extra-fine particles might be more effective to administer asthma maintenance treatments due to improved lung deposition and distribution to the distal airways (PICOT question 4).

#### Influence of adherence to ICS therapy on asthma outcomes (PICOT question 1)

Overall the observational research literature analysed here shows that poor adherence is associated with poor outcomes [38, 42–49]. However, the studies did not define thresholds of adherence which are reliably associated with improved outcomes. Thus, further analyses specifically designed to address this issue are needed. Results from observational studies suggesting that LTRA may be more effective than ICS in obtaining control in non-adherent patients (while the opposite is true for adherent patients [38]) are in line with those of a pragmatic randomized trial, which found similar effectiveness of these two drug classes in a real-life setting [90]. These data contrast with that of classical RCTs, which found greater efficacy of ICS [91]. This discrepancy might relate to differences in adherence, which is expected to be greater in classical RCTs’ patients than in real-life populations, in which it could be superior with an oral drug than with an inhaled treatment. The reluctance of some patients to take corticosteroids might also play a role.

#### Influence of smoking on asthma outcomes in patients receiving ICS therapy (PICOT question 3)

One study found that asthma control was impaired by smoking [33], which confirms previous evidence [92]. However, this study did not report differential treatment effects in patients with different smoking-related behaviours, contrary to results of previous RCTs [4] and in vitro studies on smoking-induced mechanisms of decreased sensitivity to corticosteroids [83, 84]; this discrepancy might be the consequence of intricate factors influencing the effects of maintenance therapy including adherence to treatments. In addition, the findings from this study need to be interpreted with caution due to several limitations (high drop-out rate, no adjustment nor matching, few clinical data available) that prevent elevation of the evidence quality level provided by this article. The results of another study suggest a beneficial effect of extra-fine versus fine particle ICS in smokers and ex-smokers [65]. However, although patients were matched and analyses were adjusted, differential treatment effect was only an exploratory objective of the study. Overall, this topic clearly requires additional research to disentangle the influence of smoking versus other factors modulating asthma control and treatment effects.

#### Influence of device type for ICS therapy on asthma outcomes (PICOT question 2)

Regarding PICOT question 2, the main findings from observational studies were that mixing devices in a single patient or switching devices without proper support are associated with impaired asthma control [60, 62]. This finding is especially important in the context of constrained economic resources, leading some healthcare systems to recommend treatment substitutions by less costly generic (same device) or hybrid (different device) alternatives, whenever available. Regarding direct devices comparisons, findings are contradictory; one study concluded that an MDI is associated with better outcomes than a DPI (in patients receiving an ICS/LABA combination [61]), while the other study by the same research team suggested that a breath-actuated inhaler or a DPI were more effective than a MDI (to deliver ICS) [34]. The first of these findings was rather unexpected as DPIs were developed to alleviate the need for patients to coordinate the timing of actuation and breath intake [6, 81], which is a common inhaler technique error among MDIs users [78]. It could be the result of wider patient preference for MDIs leading to increased adherence and subsequently better outcomes than for DPIs. This hypothesis could not, however, be tested in the study. The second finding could relate to improved ease-of-use and/or adherence of BAIs and DPIs (compared to MDIs); this needs to be further assessed in future studies that minimise potential confounding factors. The apparent discrepancy between these studies could also relate to differences between the open inclusion of all DPI devices in the first study, all of which have unique characteristics, compared to the restriction to a single, specific DPI in the second study [34, 61]. Again, despite the constantly growing number of available inhaler devices, each of which offers potential advantages or drawbacks as compared to their predecessors and alternatives, only a few studies have been specifically designed to compare clinical outcomes between different devices. This is only partial due to the limited number of pharmacological agents administered using different devices.

#### Influence of ICS particle size on asthma outcomes (PICOT question 3)

Several studies (most performed by a single research group) found that asthma outcomes were better in real-life patients receiving ICS or ICS-LABA combinations delivered by extra-fine rather than fine particle formulations [32, 40, 67–72]. These studies followed strict processes to minimize severity biases, including matching and adjustment strategies. However, as always in observational database research, it is not possible to totally exclude residual confounders that would be detected only if full clinical data were available. Importantly, the selection and quality assessment processes used here were subsequently applied to a recently published systematic review and metaanalysis on this topic [93].

### Strengths and limitations

This is the first initiative to develop and use a quality-assessment tool specifically designed to aid appraisal of observational asthma research. Although RELEVANT could be used in other fields, testing was performed using articles reporting results from effectiveness studies in asthma. The Task Force systematically reviewed previous quality assessment tools for observational research, selected items and revised the incorporated quality domains and items based on pilot and extended pilot testing. This provided an opportunity to improve item selection and formulation and to reduce and refine the items classification into primary and secondary items to aid inter-rater agreement.

The final literature analysis served as a validation process confirming the applicability of the tool and its relevance for quality assessment with the aim of informing guidelines and clinicians. Some limitations need to be acknowledged. First, the process could be applied only to a limited number of PICOT questions considering the high burden of each set of analyses. To limit the impact of a biased selection of questions, a consensus on prioritisation was reached not only within the Task Force but also through a poll among REG and EAACI members. Similarly, a large number of REG and EAACI members contributed to the literature analysis, to limit reviewing biases. Systematic literature appraisal is time- and resource-consuming. Ultimately the use of standardised tools such as RELEVANT should facilitate this activity but the process still requires the involvement of experienced reviewers. The limited number of high-quality real-life comparative effectiveness research studies retrieved here underlines the need for a more extensive “real-life research culture”.

### Opportunities for future research and initiatives

Comprehensive quality assessment tools are now available, not only for efficacy trials (e.g., CONSORT Statement) [94], but also for their pragmatic counterparts (CONSORT Statement extension) [95], for observational studies in epidemiology (e.g., STROBE statement) [96] and, more specifically, for pharmacoepidemiology and pharmacovigilance studies (EMA-ENCePP checklist for study protocols) [22]. Another useful initiative called SPIRIT has recently published recommendations for describing clinical trials protocols [23]. Quality criteria and minimal datasets requirements for observational studies are also the topic of the UNLOCK initiative [24]. For meta-analyses, the QUOROM (quality of reporting of meta-analysis) reporting guideline and the PRISMA (preferred reporting items for systematic reviews and meta-analyses) guidance seeks to improve the reporting of key information [19, 97]. RELEVANT now provides a quality assessment tool specifically developed for real-life effectiveness research, which can be applied to determine whether and how study results can be used to inform guidelines or clinical decision-making. It may also help guide the development of observational research protocols and study dissemination. We obviously welcome input on the RELEVANT tool from the broader community to improve its usefulness.

## Conclusions

The EAACI-REG Task Force developed and tested RELEVANT, a quality assessment tool for real-life comparative effectiveness observational research. The tool was specifically used to evaluate literature relating to PICOT questions pertinent to current evidence gaps around existing asthma interventions, but can also be used in other areas of medicine. It is hoped that the availability of this tool will assist the expansion of high-quality real-life effectiveness research in the field of respiratory medicine and allergology. Importantly, several results of the literature analysis conducted using the RELEVANT tool could lead to changes in clinical practice.

## Additional file

**Additional file 1: Table S1.** Taskforce Activity Schedule Summary. **Table S2.** Literature review search terms: used to identify a list that would include asthma observational comparative effectiveness studies. **Table S3.** Reading grid used by reviewers to summarize selected articles. **Table S4.** List of REG and EAACI contributors. **Table S5.** Literature Review Assessment Overview: All papers.
