# Supplementary material for: Quality standards in respiratory real-life effectiveness research: the REal Life EVidence AssessmeNt Tool (RELEVANT): report from the Respiratory Effectiveness Group—European Academy of Allergy and Clinical Immunology Task Force
Source: Clin Transl Allergy. 2019 Mar 27;9:20. doi: 10.1186/s13601-019-0255-x (PMC6436229; doi:10.1186/s13601-019-0255-x)
Supplement: Supplementary file 1 — Additional file 1: Table S1. Taskforce Activity Schedule Summary. Table S2. Literature review search terms: used to identify a list that would include asthma observational comparative effectiveness studies. Table S3. Reading grid used by reviewers to summarize selected articles. Table S4. List of REG and EAACI contributors. Table S5. Literature Review Assessment Overview: All papers. [file 13601_2019_255_MOESM1_ESM.docx]

**ELECTRONIC SUPPLEMENTARY MATERIAL**

Quality standards in asthma comparative effectiveness research: a Respiratory Effectiveness Group – European Academy of Allergy and Clinical Immunology Task Force report

**Table S1. Taskforce Activity Schedule Summary**

| **ACTIVITY** | **START DATE** | **END DATE** |
| --- | --- | --- |
| **Proposal submitted** | September 2013 |  |
| **Taskforce approved** | November 2013 |  |
| **Taskforce group formalised** | January 2014 |  |
| **Planning** | January 2014 | April 2014 |
| **Literature review, PICOT Question Identification (and literature refinement)** | May 2014 | March 2015 |
| **Quality assessment tool development** | May 2014 | January 2015 |
| **Taskforce pilot testing** | February 2015 | May 2015 |
| **Assessment of inter-rater variability** | June 2015 | September 2015 |
| **Quality assessment of PICOT literature** | October 2015 | March 2016 |
| **Results synthesis & report writing** | March 2016 | April 2016 |
| **Results presentation & discussion** | • April 2016: REG Summit  • June 2016: EAACI Conference |  |
| **Results publication** | Q3 2016 |  |

**Literature search methodology**

Two databases were consulted to conduct the literature review: MEDLINE and EMBASE.

The search was undertaken in two phases – an initial phase and an extension phase: the i**nitial phase** reviewed the literature published in the ten-year period January 2004 to December 2014, and was c**onducted:** 13 January 2015. As the quality assessment tool was finalized in autumn 2015 and the literature review commenced in December 2015, a one-year extension period was added on 9 December 2015 to the literature review to ensure it included all current literature.

The following sensitive, but not specific search terms (and algorithms) were used for the review of the literature:

**Table S2. Literature review search terms: used to identify a list that would include asthma observational comparative effectiveness studies**

| [**# ▲**](http://ovidsp.tx.ovid.com/sp-3.13.1a/ovidweb.cgi?&S=FKACFPLAIJDDLEFENCLKDFLBPDIFAA00&Sort+Sets=descending) | **SEARCH TERM** |
| --- | --- |
|  |  |
| 1 | Comparative studies/ |
| 2 | Comparative effectiveness/ |
| 3 | Comparative effectiveness research/ |
| 4 | (Comparative adj effectiveness).tw. |
| 5 | Follow up studies/ |
| 6 | Prospective studies/ |
| 7 | Prospective$.tw. |
| 8 | Retrospective studies/ |
| 9 | Retrospective$.tw. |
| 10 | Cohort studies/ |
| 11 | Cohort.tw. |
| 12 | (compare$ or compara$).tw. |
| 13 | Compared.tw. |
| 14 | Case control studies/ |
| 15 | (Observational adj (study or studies)).tw. |
| 16 | Treatment Outcome/ |
| 17 | Database.tw. |
| 18 | or/1-17 |
| 19 | Asthma/ |
| 20 | Inhaled corticosteroid$.tw. |
| 21 | Azithromycin.tw. |
| 22 | Macrolide$.tw. |
| 23 | or/20-22 |
| 24 | 18 and 19 and 23 |
| 25 | Randomized controlled trial/ |
| 26 | Random allocation/ |
| 27 | 25 or 26 |
| 28 | 24 not 27 |
| **29** | **limit 28 to (English language and humans and year="2004 -Current")** |

Papers were reviewed initially to identify and remove any duplicates found (i.e. retrieved via both the MEDLINE and EMBASE searches) and then further filtered based on their fulfilment of the following exclusion criteria:

- Conference proceedings and/or abstract only
- Not an asthma study (e.g. COPD, Allergic rhinitis)
- Not a comparative effectiveness study, including:
  - - Literature review
    - Clinical trial
    - Case study
    - Cross-sectional survey
    - Cohort study

**Table S3: reading grid used by reviewers to summarize selected articles**

| **REFERENCE** |  |
| --- | --- |
| **FUNDING** |  |
| **STUDY DESIGN** |  |
| **INCLUDED POPULATION** |  |
| **SETTING, TIME FRAME**  **GEOGRAPHIC AREA** |  |
| **COMPARED ARMS  (IF APPLICABLE)** |  |
| **DURATION OF FOLLOW UP** |  |
| **PRIMARY OBJECTIVE, OUTCOME AND ANALYSIS** |  |
| **OTHER OBJECTIVES, ANALYSES AND RESULTS** |  |
| **PRIMARY RESULTS** |  |
| **OTHER RESULTS** |  |
| **POSSIBLE BIASES** |  |
| **CONCLUSION OF THE AUTHORS** |  |
| **STRENGTH OF CONCLUSION  (rater opinion)** |  |

**Table S4. List of REG and EAACI contributors**

| **MEMBER** | **COUNTRY** | **CONTRIBUTION** |
| --- | --- | --- |
| **Aileen Wang** | The Philippines | QR |
| **Alan Kaplan** | Canada | QR, OPR |
| **Andrew McIvor** | Canada | QR |
| **Anjan Nibber** | UK | QR, OPR |
| **Antoine Magnan** | France | QR |
| **Anu Kemppinen** | UK | QR |
| **Arzu Bakırtaş** | Turkey | QATT |
| **Bakhtiyor Khalikulov** | UK | QR |
| **Bernardino Alcazar Navarrette** | Spain | QATT, QR |
| **Brett McQueen** | USA | QR, OPR |
| **Chong Kim** | USA | QR, OPR |
| **Christina Callan** | UK | QR |
| **David Halpin** | UK | QATT |
| **Dmitris Mitsias** | Greece | QR |
| **Emilio Pizzichini** | Brazil | QR |
| **Enrico Heffler** | Italy | QATT, QR |
| **George Guibas** | UK | QR, OPR |
| **George Konstantinou** | Greece | QR |
| **Helen Reddell** | Australia | QATT, QR |
| **Ioannis Taslos** | Greece | QR, OPR |
| **Janwillem Kocks** | The Netherlands | QR |
| **Jennifer Quint** | UK | OPR |
| **Joan Soriano** | Spain | QR |
| **Job van Boven** | The Netherlands | QR |
| **Joergen Vestbo** | Denmark | QATT |
| **John Blakey** | UK | QR, OPR |
| **Juan José Soler Cataluna** | Spain | QATT |
| **Katia Verhamme** | Belgium | QR |
| **Kostas Kostikas** | Greece | QATT, QR |
| **Lars Olaf Cardell** | Sweden | QR |
| **Laurent Laforest** | France | QATT |
| **Laurie Pahus** | France | OPR |
| **Elizabeth Hillyer** | USA | QR |
| **Ludger Klimek** | Germany | QATT, QR |
| **Luis Caraballo** | Colombia | QATT |
| **Lynn Josephs** | UK | QR |
| **Manon Laforest** | France | QATT |
| **Maria Ospina** | Canada | QR |
| **Marjan Kerkoff** | The Netherlands | QR |
| **Matte Bonini** | Italy | QATT |
| **Melanie Whittington** | USA | QR, OPR |
| **Mihaela Stefan** | USA | QR, OPR |
| **Ming-Cheng Chan** | China | QR |
| **Mohsen Sadatsafavi** | Canada | QR, OPR |
| **Ömer Kalayci** | Greece | QATT |
| **Paraskevi Maggina** | Greece | QR, OPR |
| **Piyameth Dilokthomsakul** | Malaysia | QATT, QR |
| **Ronald Dandurand** | Canada | QR, OPR |
| **Sam Sonnappa** | India | QR, OPR |
| **Shawna Tan** | Singapore | QR |
| **Simon van Rysewyk** | Singapore | QR, OPR |
| **Sinthia Bosnic-Anticevich** | Australia | QATT |
| **Steve Turner** | UK | QR, OPR |
| **Theresa Guilbert** | USA | QR, OPR |
| **Yee Vern Yong** | Malaysia | QATT, QR |

**Table S5. Literature Review Assessment Overview: All papers**

**Papers categorized by PICOT question and quality rating**

| **#** | **PAPER DETAILS** | **SUFFICIENTLY HIGH QUALITY** | | **INSUFFICIENTLY HIGH QUALITY** | **PRIMARY QUALITY LIMITATIONS*** |  |  |
| --- | --- | --- | --- | --- | --- | --- | --- |
| **PICOT 1: “ADHERENCE TO ICS THERAPY”; 24 PAPERS** | | | | | |  |  |
| 1 | Sadatsafavi M, Lynd LD, Marra CA, FitzGerald JM. Dispensation of long-acting β agonists with or without inhaled corticosteroids, and risk of asthma-related hospitalisation: a population-based study.  *Thorax*. **2014**;69(4):328-34 | ✔ | | ✖ | NA |  |  |
| 2 | Friedman HS, Navaratnam P, McLaughlin J. Adherence and asthma control with mometasone furoate versus fluticasone propionate in adolescents and young adults with mild asthma. J Asthma. **2010**;47(9):994-1000. | ✔ | | ✖ | NA |  |  |
| 3 | Campbell JD, Allen-Ramey F, Sajjan SG, Maiese EM, Sullivan SD. Increasing pharmaceutical copayments: impact on asthma medication utilization and outcomes. Am J Manag Care. **2011**;17(10):703-10. | ✔ | | ✖ | NA |  |  |
| 4 | Tan H, Sarawate C, Singer J, Elward K, Cohen RI, Smart BA, Busk MF, Lustig J, O'Brien JD, Schatz M. Impact of asthma controller medications on clinical, economic, and patient-reported outcomes. Mayo Clin Proc. **2009**;84(8):675-84 | ✔ | | ✖ | NA |  |  |
| 5 | Williams LK, Pladevall M, Xi H, Peterson EL, Joseph C, Lafata JE, Ownby DR, Johnson CC. Relationship between adherence to inhaled corticosteroids and poor outcomes among adults with asthma. J Allergy Clin Immunol. **2004**;114(6):1288-93 | ✔ | | ✖ | NA |  |  |
| 6 | Taegtmeyer AB, Steurer-Stey C, Price DB, Wildhaber JH, Spertini F, Leuppi JD. Predictors of asthma control in everyday clinical practice in Switzerland.  Curr Med Res Opin. **2009**;25(10):2549-55 | ✔ | | ✖ | NA |  |  |
| 7 | Laforest L, Licaj I, Devouassoux G, Chatté G, Belhassen M, Van Ganse E, Chamba G. Relative exposure to controller therapy and asthma exacerbations: a validation study in community pharmacies. Pharmacoepidemiol Drug Saf. **2014**;23(9):958-64. | ✔ | | ✖ | NA |  |  |
| 8 | Laforest L, Licaj I, Devouassoux G, Chatte G, Martin J, Van Ganse E. Asthma drug ratios and exacerbations: claims data from universal health coverage systems. Eur Respir J. **2014**;43(5):1378-86 | ✔ | | ✖ | NA |  |  |
| 9 | Björnsdóttir US, Sigurðardóttir ST, Jonsson JS, Jonsson M, Telg G, Thuresson M, Naya I, Gizurarson S. Impact of changes to reimbursement of fixed combinations of inhaled corticosteroids and long-acting β2-agonists in obstructive lung diseases: a population-based, observational study. Int J Clin Pract. 2014;68(7):812-9. | ✔ | | ✖ | NA | |  |
| 10 | Lee T, Kim J, Kim S, Kim K, Park Y, Kim Y, Lee YS, Kwon HS, Kim SH, Chang YS, Cho YS, Jang AS, Park JW, Nahm DH, Yoon HJ, Cho SH, Cho YJ, Choi BW, Moon HB, Kim TB; COREA study group. Risk factors for asthma-related healthcare use: longitudinal analysis using the NHI claims database in a Korean asthma cohort. PLoS One. **2014**;9(11):e112844 | ✖ | | ✔ | Both reviewers: 2.2  1 reviewer: 3.1, 3.2, 4.2, 5.1, 7.1  **1 ‘agreed’ limitation; *unique limitations identified*** | |  |
| 11 | Taylor A, Chen LC, Smith MD. Adherence to inhaled corticosteroids by asthmatic patients: measurement and modelling. Int J Clin Pharm. **2014**;36(1):112-9. | ✖ | | ✔ | Both reviewers: 2.2, 4.2  1 reviewer: 3.2  **2 ‘agreed’ limitations; *unique limitations identified*** | |  |
| *12* | *Rust G, Zhang S, Reynolds J. Inhaled corticosteroid adherence and emergency department utilization among Medicaid-enrolled children with asthma. J Asthma.* ***2013****;50(7):769-75.* | *✖* | | *✔* | *Both reviewers: 2.2*  *1 reviewer: 4.1, 4.2, 5.1, 7.1*  ***1 ‘agreed’ limitation; 4 unique limitations identified*** | |  |
| 13 | Elkout H, Helms PJ, Simpson CR, McLay JS. Adequate levels of adherence with controller medication is associated with increased use of rescue medication in asthmatic children. PLoS One. **2012**;7:e39130 | ✖ | | ✔ | *Both reviewers: 2.2, 4.2*  ***2 ‘agreed’ limitations*** | |  |
| 14 | Herndon JB, Mattke S, Evans Cuellar A, Hong SY, Shenkman EA . Anti-inflammatory medication adherence, healthcare utilization and expenditures among Medicaid and children's health insurance program enrollees with asthma. Pharmacoeconomics. **2012**;30(5):397-412 | ✖ | | ✔ | *Both reviewers: 2.2*  *1 reviewer: 4.2*  ***1 ‘agreed’ limitation; 1 unique limitation identified*** | |  |
| *15* | *Giraud V, Allaert FA, Roche N. Inhaler technique and asthma: feasability and acceptability of training by pharmacists. Respir Med.* ***2011****;105(12):1815-22* | *✖* | | *✔* | *Both reviewers: 4.1, 5.1*  *1 reviewer: 4.2*  ***2 ‘agreed’ limitations; 1 unique limitation identified*** | |  |
| 16 | Sawicki GS, Strunk RC, Schuemann B, Annett R, Weiss S, Fuhlbrigge AL; Childhood Asthma Management Program Research Group. Patterns of inhaled corticosteroid use and asthma control in the Childhood Asthma Management Program Continuation Study. Ann Allergy Asthma Immunol. **2010**;104(1):30-5 | | ✖ | ✔ | *Both reviewers: 5.1*  *1 reviewer: 4.1, 6.1, 6.2*  ***1 ‘agreed’ limitations;  3 unique limitations identified*** | | |
| 17 | Bukstein DA, Murphy KR, Katz LM, Ramachandran S, Doyle KK, Stern LS. Outcomes Among a Young Population of Pediatric Asthma Patients Using Controller Therapies: Results from a Retrospective Database Analysis. Pediatric Asthma, Allergy & Immunology. **2007**;20(4):211-223 | | ✖ | ✔ | *Both reviewers: 4.2, 7.1*  *1 reviewer: 3.1*  ***2 ‘agreed’ limitations;  1 unique limitation identified*** | | |
| *18* | *Lasmar L, Camargos P, Champs NS, Fonseca MT, Fontes MJ, Ibiapina C, Alvim C, Moura JA. Adherence rate to inhaled corticosteroids and their impact on asthma control. Allergy.* ***2009****;64(5):784-9* | | *✖* | *✔* | *Both reviewers: 7.1*  *1 reviewer: 4.1, 4.2*  ***1 ‘agreed’ limitation;  2 unique limitation identified*** | | |
| 19 | Santos Pde M, D'Oliveira A Jr, Noblat Lde A, Machado AS, Noblat AC, Cruz AA. Predictors of adherence to treatment in patients with severe asthma treated at a referral center in Bahia, Brazil. J Bras Pneumol. **2008**;34(12):995-1002 | | ✖ | ✔ | Both reviewers: 7.1  1 reviewer: 4.2  **1 ‘agreed’ limitation;  1 unique limitation identified** | | |
| 20 | Klok T, Kaptein AA, Duiverman EJ, Brand PL. It's the adherence, stupid (that determines asthma control in preschool children)! Eur Respir J. **2014**;43(3):783-91 | | ✖ | ✔ | Both reviewers: 4.1 | | |
| 21 | Guest JF, Davie AM, Ruiz FJ, Greener MJ. Switching asthma patients to a once-daily inhaled steroid improves compliance and reduces healthcare costs. Prim Care Respir J. **2005**;14(2):88-98 | | ✖ | ✔ | 1 reviewer: 4.1, 4.2, 5.1  **3 unique limitation identified** | | |
| 22 | Price D, Chisholm A, Hillyer EV, Burden A, von Ziegenweidt J, Svedstater H, Dale P. Effect of Inhaled Corticosteroid Therapy Step-Down and Dosing Regimen on Measures of Asthma Control. J Aller Ther. **2013**(4):126 | | ✖ | ✔ | Both reviewers: 7.1  **1 ‘agreed’ limitation** | | |
| 23 | Klok T, Kaptein AA, Duiverman EJ, Brand PL. Long-term adherence to inhaled corticosteroids in children with asthma: Observational study. Respir Med. **2015**;109(9):1114-9 | | ✖ | ✔ | 1 reviewer: 2.2, 4.2 **2 unique limitation identified** | | |
| 24 | Dalcin Pde T, Grutcki DM, Laporte PP, Lima PB, Viana VP, Konzen GL, Menegotto SM, Fonseca MA, Pereira RP. Impact of a short-term educational intervention on adherence to asthma treatment and on asthma control. J Bras Pneumol. **2011**;37(1):19-27 | | ✖ | ✔ | Both reviewers: 4.1, 5.1  1 reviewer: 2.2, 4.2, 6.1, 7.1  **2 ‘agreed’ limitations;  4 unique limitation identified** | | |

| **#** | **PAPER DETAILS** | **SUFFICIENTLY HIGH QUALITY** | **INSUFFICIENTLY HIGH QUALITY** | **PRIMARY QUALITY LIMITATIONS*** |
| --- | --- | --- | --- | --- |
| **PICOT 2: “DEVICE TYPE”; 7 PAPERS** | | | | |
| 1 | Price D, Chrystyn H, Kaplan A, Haughney J, Román-Rodríguez M, Burden A, Chisholm A, Hillyer EV, von Ziegenweidt J, Ali M, van der Molen T. Effectiveness of same versus mixed asthma inhaler devices: a retrospective observational study in primary care. Allergy Asthma Immunol Res. **2012**;4(4):184-91 | ✔ | ✖ | NA |
| 2 | Price D, Roche N, Christian Virchow J, Burden A, Ali M, Chisholm A, Lee AJ, Hillyer EV, von Ziegenweidt J. Device type and real-world effectiveness of asthma combination therapy: an observational study. Respir Med. **2011**;105(10):1457-66. | ✔ | ✖ | NA |
| 3 | Price D, Haughney J, Sims E, Ali M, von Ziegenweidt J, Hillyer EV, Lee AJ, Chisholm A, Barnes N. Effectiveness of inhaler types for real-world asthma management: retrospective observational study using the GPRD. J Asthma Allergy. **2011**;4:37-47. | ✔ | ✖ | NA |
| 4 | Thomas M, Price D, Chrystyn H, Lloyd A, Williams AE, von Ziegenweidt J. Inhaled corticosteroids for asthma: impact of practice level device switching on asthma control. BMC Pulm Med. **2009**;9:1. doi: 10.1186/1471-2466-9-1. | ✔ | ✖ | NA |
| 5 | Voshaar T, Kostev K, Rex J, Schröder-Bernhardi D, Maus J, Munzel U. A retrospective database analysis on persistence with inhaled corticosteroid therapy: comparison of two dry powder inhalers during asthma treatment in Germany. Int J Clin Pharmacol Ther. **2012**;50(4):257-64. | ✔ | ✖ | NA |
| 6 | Price D, Thomas V, von Ziegenweidt J, Gould S, Hutton C, King C. Switching patients from other inhaled corticosteroid devices to the Easyhaler(®): historical, matched-cohort study of real-life asthma patients. J Asthma Allergy. **2014**;7:31-51 | ✔ | ✖ | NA |
| 7 | Kemp L, Haughney J, Barnes N, Sims E, von Ziegenweidt J, Hillyer EV, Lee AJ, Chisholm A, Price D. Cost-effectiveness analysis of corticosteroid inhaler devices in primary care asthma management: A real world observational study. Clinicoecon Outcomes Res. **2010**;2:75-85 | ✖ | ✔ | Both reviewers: TBC  1 reviewer: 4.1  **TBC ‘agreed’ limitations;  1 unique limitation identified** |

| **#** | **PAPER DETAILS** | **SUFFICIENTLY HIGH QUALITY** | | **INSUFFICIENTLY HIGH QUALITY** | **PRIMARY QUALITY LIMITATIONS*** |
| --- | --- | --- | --- | --- | --- |
| **PICOT 3: “SMOKING ASTHMATICS”; 3 PAPERS** | | | | | |
| 1 | Brusselle G, Peché R, Van den Brande P, Verhulst A, Hollanders W, Bruhwyler J. Real-life effectiveness of extrafine beclometasone dipropionate/formoterol in adults with persistent asthma according to smoking status. Respir Med. **2012**;106(6):811-9 | | ✔ | ✖ | NA |
| 2 | Roche N, Postma DS, Colice G, Burden A, Guilbert TW, Israel E, Martin RJ, van Aalderen WM, Grigg J, Hillyer EV, von Ziegenweidt J, Price DB. Differential effects of inhaled corticosteroids in smokers/ex-smokers and nonsmokers with asthma. Am J Respir Crit Care Med. **2015**;191(8):960-4. | | ✔ | ✖ | NA |
| *3* | *Telenga ED, Kerstjens HA, Ten Hacken NH, Postma DS, van den Berge M. Inflammation and corticosteroid responsiveness in ex-, current- and never-smoking asthmatics. BMC Pulm Med.* ***2013****;22;13:58* | | *✖* | *✔* | *1 reviewer: 2.1, 3.2, 4.1, 4.2, 5.1*  ***4 unique limitations identified*** |
| **PICOT 4: “SMALL AIRWAYS MANAGEMENT; ICS PARTICLE SIZE”** | | | | | |
| 1 | van Aalderen WM, Grigg J, Guilbert TW, Roche N, Israel E, Martin RJ, Colice G, Postma DS, Hillyer EV, Burden A, Thomas V, von Ziegenweidt J, Price D. Small-particle Inhaled Corticosteroid as First-line or Step-up Controller Therapy in Childhood Asthma. J Allergy Clin Immunol Pract. **2015**;3(5):721-31 | | ✔ | ✖ | NA |
| 2 | Martin RJ, Price D, Roche N, Israel E, van Aalderen WM, Grigg J, Postma DS, Guilbert TW, Hillyer EV, Burden A, von Ziegenweidt J, Colice G. Cost-effectiveness of initiating extrafine- or standard size-particle inhaled corticosteroid for asthma in two health-care systems: a retrospective matched cohort study. NPJ Prim Care Respir Med. **2014**;24:14081 | | ✔ | ✖ | NA |
| 3 | Colice G, Martin RJ, Israel E, Roche N, Barnes N, Burden A, Polos P, Dorinsky P, Hillyer EV, Lee AJ, Chisholm A, von Ziegenweidt J, Barion F, Price D. Asthma outcomes and costs of therapy with extrafine beclomethasone and fluticasone. J Allergy Clin Immunol. **2013**;132(1):45-54 | | ✔ | ✖ | NA |
| 4 | Price D, Thomas M, Haughney J, Lewis RA, Burden A, von Ziegenweidt J, Chisholm A, Hillyer EV, Corrigan CJ. Real-life comparison of beclometasone dipropionate as an extrafine- or larger-particle formulation for asthma. Respir Med. **2013**;107(7):987-1000 | | ✔ | ✖ | NA |

| **#** | | **PAPER DETAILS** | **SUFFICIENTLY HIGH QUALITY** | | **INSUFFICIENTLY HIGH QUALITY** | **PRIMARY QUALITY LIMITATIONS*** |
| --- | --- | --- | --- | --- | --- | --- |
| 5 | Price D, Martin RJ, Barnes N, Dorinsky P, Israel E, Roche N, Chisholm A, Hillyer EV, Kemp L, Lee AJ, von Ziegenweidt J, Colice G. Prescribing practices and asthma control with hydrofluoroalkane-beclomethasone and fluticasone: a real-world observational study. J Allergy Clin Immunol. **2010**;126(3):511-8.e1-10 | | | ✔ | ✖ | NA |
| 6 | Allegra L, Cremonesi G, Girbino G, Ingrassia E, Marsico S, Nicolini G, Terzano C; PRISMA (PRospectIve Study on asthMA control) Study Group. Real-life prospective study on asthma control in Italy: cross-sectional phase results. Respir Med. **2012**;106(2):205-14 | | | ✔ | ✖ | NA |
| 7 | Barnes N, Price D, Colice G, Chisholm A, Dorinsky P, Hillyer EV, Burden A, Lee AJ, Martin RJ, Roche N, von Ziegenweidt J, Israel E. Asthma control with extrafine-particle hydrofluoroalkane-beclometasone vs. large-particle chlorofluorocarbon-beclometasone: a real-world observational study. Clin Exp Allergy. **2011**;41(11):1521-32 | | | ✔ | ✖ | NA |
| 8 | Price D, Small I, Haughney J, Ryan D, Gruffydd-Jones K, Lavorini F, Harris T, Burden A, Brockman J, King C, Papi A. Clinical and cost effectiveness of switching asthma patients from fluticasone-salmeterol to extra-fine particle beclometasone-formoterol: a retrospective matched observational study of real-world patients. Prim Care Respir J. **2013**;22(4):439-48 | | | ✔ | ✖ | NA |
| 9 | Müller V, Gálffy G, Eszes N, Losonczy G, Bizzi A, Nicolini G, Chrystyn H, Tamási L. Asthma control in patients receiving inhaled corticosteroid and long-acting beta2-agonist fixed combinations. A real-life study comparing dry powder inhalers and a pressurized metered dose inhaler extrafine formulation. BMC Pulm Med. **2011**;11:40 | | | ✖ | ✔ | Both reviewers: TBC  1 reviewer: TBC  **TBC ‘agreed’ limitations;  TBC unique limitation identified** |
| 10 | Popov TA, Petrova D, Kralimarkova TZ, Ivanov Y, Popova T, Peneva M, Odzhakova T, Ilieva Y, Yakovliev P, Lazarova T, Georgiev O, Hodzhev V, Hodzheva E, Staevska MT, Dimitrov VD. Real life clinical study design supporting the effectiveness of extra-fine inhaled beclomethasone/formoterol at the level of small airways of asthmatics. Pulm Pharmacol Ther. 2013;26(6):624-9 | | | ✖ | ✔ | 1 reviewer: 2.1, 2.2, 3.2, 4.1, 4.2, 5.1, 7.1  **7 unique limitations identified** |
| 11 | Paggiaro P, Patel S, Nicolini G, Pradelli L, Zaniolo O, Papi A. Stepping down from high dose fluticasone/salmeterol to extrafine BDP/F in asthma is cost-effective. Respir Med. **2013**;107(10):1531-7 | | | ✖ | ✔ | 1 reviewer: 2.2, 3.1, 4.1, 6.2  **4 unique limitations identified** |
| **#** | **PAPER DETAILS** | | | **SUFFICIENTLY HIGH QUALITY** | **INSUFFICIENTLY HIGH QUALITY** | **PRIMARY QUALITY LIMITATIONS*** |
| 12 | Israel E, Roche N, Martin RJ, Colice G, Dorinsky PM, Postma DS, Guilbert TW, van Aalderen WM, Grigg J, Hillyer EV, Burden A, von Ziegenweidt J, Thomas V, Price DB. Increased Dose of Inhaled Corticosteroid versus Add-On Long-acting β-Agonist for Step-Up Therapy in Asthma. Ann Am Thorac Soc. **2015**;12(6):798-806 | | | ✖ | ✔ | Both reviewers: TBC  1 reviewer: 3.2, 5.1, 7.1  **TBC ‘agreed’ limitations;  3 unique limitation identified** |

*Italic text indicates papers that required a third adjudicating review.*

*Primary limitations are counted across both “negative” reviews each paper received, split by those identified as limitations by 1 or both reviewers*

**Limitation number related to field with in the literature quality assessment tool*

***Key: Primary quality criteria***

***1. Background: 1.1.*** *Clearly stated research question;*

***2. Design: 2.1*** *Population defined* ***2.2.*** *Comparison groups defined;*

***3. Measures: 3.1.*** *If relevant, is exposure (e.g. treatment) defined* ***3.2.*** *Primary Outcomes defined;*

***4. Analysis:*** *4.1. Potential confounders are addressed* ***4.2.*** *Study groups are compared at baseline;*

***5. Results. 5.1*** *Results are clearly presented for primary endpoints as well as confounders;*

***6. Discussion and Interpretation: 6.1.*** *Results consistent with known information or, if not, an explanation is provided;* ***6.2.*** *Clinical relevance of the results is discussed;*

***7. Conflicts of Interest: 7.1.*** *Potential conflicts of interest, including study funding, are stated*
